# Surface-Enhanced Raman Scattering Combined with Machine Learning for Rapid and Sensitive Detection of Anti-SARS-CoV-2 IgG

**DOI:** 10.3390/bios14110523

**Published:** 2024-10-29

**Authors:** Thais de Andrade Silva, Gabriel Fernandes Souza dos Santos, Adilson Ribeiro Prado, Daniel Cruz Cavalieri, Arnaldo Gomes Leal Junior, Flávio Garcia Pereira, Camilo A. R. Díaz, Marco Cesar Cunegundes Guimarães, Servio Túlio Alves Cassini, Jairo Pinto de Oliveira

**Affiliations:** 1 Morphology Department, Federal University of Espirito Santo, Av Marechal Campos, 1468, Vitória 29040-090, ES, Brazil; thais.a.silva@edu.ufes.br (T.d.A.S.); gfss.quimica@gmail.com (G.F.S.d.S.); marco.guimaraes@ufes.br (M.C.C.G.); 2 Federal Institute of Espírito Santo, Campus Serra, Serra 29173-087, ES, Brazil; adilsonrp@ifes.edu.br (A.R.P.); daniel.cavalieri@ifes.edu.br (D.C.C.); flavio.garcia@ifes.edu.br (F.G.P.); 3 Telecommunications Laboratory, Electrical Engineering Department, Federal University of Espírito Santo (UFES), Av Fernando Ferrari 514, Vitória 29075-910, ES, Brazil; arnaldo.leal@ufes.br (A.G.L.J.); camilo.diaz@ufes.br (C.A.R.D.); 4 Center of Research, Innovation and Development of Espirito Santo, Ladeira Eliezer Batista, Cariacica 29140-130, ES, Brazil; servio.cassini@ufes.br

**Keywords:** gold nanoparticles, multivariate analysis, SERS, machine learning

## Abstract

This work reports an efficient method to detect SARS-CoV-2 antibodies in blood samples based on SERS combined with a machine learning tool. For this purpose, gold nanoparticles directly conjugated with spike protein were used in human blood samples to identify anti-SARS-CoV-2 antibodies. The comprehensive database utilized Raman spectra from all 594 blood serum samples. Machine learning investigations were carried out using the Scikit-Learn library and were implemented in Python, and the characteristics of Raman spectra of positive and negative SARS-CoV-2 samples were extracted using the Uniform Manifold Approximation and Projection (UMAP) technique. The machine learning models used were k-Nearest Neighbors (kNN), Support Vector Machine (SVM), Decision Trees (DTs), logistic regression (LR), and Light Gradient Boosting Machine (LightGBM). The kNN model led to a sensitivity of 0.943, specificity of 0.9275, and accuracy of 0.9377. This study showed that combining Raman spectroscopy and a machine algorithm can be an effective diagnostic method. Furthermore, we highlighted the advantages and disadvantages of each algorithm, providing valuable information for future research.

## 1. Introduction

The COVID-19 pandemic caused by the SARS-CoV-2 virus has had a profound impact on the world, resulting in morbidity and mortality. According to the World Health Organization (WHO), as of February 2024, there have been more than 770 million confirmed cases of COVID-19, leading to approximately 7 million deaths [1,2]. These numbers highlight the critical need to understand the global clinical course of this disease [3]. To mitigate the effects of the pandemic, early diagnosis is essential for initiating the most effective therapy and managing the spread of infections. Therefore, developing tools for continuous and reliable monitoring of COVID-19 is crucial [4].

The gold standard for detecting SARS-CoV-2 is real-time reverse transcriptase polymerase chain reaction (RT-PCR). However, RT-PCR has several limitations, including long assay times, high costs, the need for skilled personnel, and specialized infrastructure [5]. To address these drawbacks, alternative methods have been developed, such as electrochemical sensors [6,7,8,9], lateral flow immunosensors [10], luminescent sensors [11], and spectroscopy sensors [12,13,14,15,16,17,18]. Among these, surface-enhanced Raman scattering (SERS) stands out as a highly promising spectroscopic technique due to its portability and potential for large-scale analysis. SERS relies on the use of plasmonic materials, such as gold (AuNPs) and silver nanoparticles (AgNPs), which enhance the Raman signal [19]. These metals can conjugate with antigens and antibodies, facilitating the development of strategies to improve the sensitivity and selectivity of Raman spectroscopy [20]. Zhang et al. (2021) developed a sensor for the SARS-CoV-2 spike protein in untreated saliva using a substrate of AgNPs covered with 4-mercaptobenzoic acid (4-MBA) as a Raman reporter and binding with the SARS-CoV-2 spike antibody. This material detects SARS-CoV-2 spike protein at a concentration of 6.07 fg mL^−1^ in untreated saliva [16].

In addition, starting in the 2010s, many computational advancements emerged in pattern recognition applied to images. Among these, the integration of multivariate analysis with machine learning has proven particularly useful, enabling the interpretation of complex datasets and the training of neural networks to deliver diagnostic results in point-of-care settings [5,14,15,17,21]. This combination of advanced algorithms with diagnostic tools allows for more rapid and accurate analysis of biological samples. In this sense, de Almeida et al. (2022) utilized Matrix-Assisted Laser Desorption/Ionization Fourier-transform Ion Cyclotron Resonance Mass Spectrometry (MALDI FT-ICR MS) to develop COVID-19 diagnostic screening. Saliva samples from 97 and 52 patients positive and negative for SARS-CoV-2 were analyzed using this method. A Support Vector Machine (SVM) was employed to analyze all mass spectra, resulting in an accuracy of 100% for the calibration group and 95.6% for the test group [14]. Similarly, Nascimento et al. (2022) used multivariate analysis combined with Mid-Infrared (MIR) data to detect COVID-19. The authors analyzed 237 saliva samples, of which 138 were positive by RT-PCR. Multivariate analyses were performed using unsupervised and supervised methods, achieving good validation performance with 85% accuracy, 93% sensitivity, and 83% specificity [15]. Another study that can be highlighted is Pazin et al. (2024), who designed a SERS substrate based on gold nanoislands conjugated with 4-aminothiophenol (Raman reporter) and an anti-SARS-CoV-2 antibody. The authors studied the behavior of the substrate only in phosphate buffer solution (PBS) in the presence and absence of recombinant human SARS-CoV-2 spike glycoprotein S1 and with p53 protein used as an interferent. The machine learning system used was an SVM associated with linear discriminant analysis and logistic regression, which achieved accuracies of 96–100% [17].

However, despite the promising potential of combining SERS with machine learning, several challenges remain. One of the primary limitations is the need for extensive and diverse datasets to adequately train machine learning models. In the case of antibody detection, the variability between patient samples and potential interference from other proteins present in blood serum can reduce the overall accuracy and reliability of the method. Additionally, the reproducibility of SERS signals can be affected by factors such as nanoparticle aggregation and the uniformity of substrate preparation, which may hinder its practical application in clinical settings. Addressing these limitations is essential to fully harness the capabilities of SERS and machine learning for accurate diagnostics.

In this study, we sought to overcome these challenges by integrating machine learning techniques with SERS data to rapidly and accurately analyze SARS-CoV-2 antibodies in authentic blood serum samples. By utilizing colloidal AuNPs conjugated with the SARS-CoV-2 spike protein, we exploited the SERS effect to differentiate between positive and negative samples.

The procedure to distinguish positive samples involved collecting SERS spectra from 594 blood serum samples, followed by baseline correction, normalization, and feature extraction using UMAP for dimensionality reduction. Several machine learning models were evaluated, with k-Nearest Neighbors (kNN) showing the best performance. The kNN model achieved an accuracy of 93.77%, sensitivity of 94.31%, and precision of 96.05%, effectively distinguishing positive SARS-CoV-2 antibody samples. These metrics demonstrate the method’s robustness and reliability for diagnostic applications.

## 2. Materials and Methods

### 2.1. Materials and Reagents

Tetrachloroauric acid (Sigma Aldrich, 520918, Missouri, USA), sodium citrate (Dinâmica, 6132043, São Paulo, Brazil), recombinant SARS-CoV-2 spike protein (Abcam, ab273063) were used. All solutions were prepared using ultrapure water with a resistivity of 18.2 MΩ.cm, obtained using the Synergy^®^ Water Purification System (Mili-Q, Merck, Darmstadt, Germany).

### 2.2. Synthesis of Gold Nanoparticles

AuNPs were synthesized using the method of Oliveira et al. (2020) [22] adapted for the Monowave 50 synthesis reactor (Anton-Paar, Graz, Austria). The method used a 15:1 ratio of 2.5 mM tetrachloroauric acid and 1% sodium citrate. In the reactor, the HAuCl_4_ solution was subjected to a 1 min cycle at 100 °C under stirring at 400 rpm and cooled to 70 °C at the end of the cycle. Subsequently, 1% sodium citrate was added to the reaction, which was cycled for 15 min at 100 °C and stirred at 400 rpm. The solution was then cooled to room temperature at the end of the cycle.

### 2.3. Bioconjugation of AuNPs

Protein bioconjugation with AuNPs was carried out using 0.2 mg mL^−1^ of the recombinant spike glycoprotein (ab273063). The protein load was standardized for bioconjugation using the gold number technique [23]. In summary, the protein was serially diluted from 100 μg·mL^−1^ to 1.56 μg·mL^−1^ using ultrapure water in a 96-well plate. Equal volumes of AuNPs were then added to each well, and the conjugation was allowed to proceed for 20 min at 25 °C. Subsequently, 10 μL of a 10% NaCl solution was introduced to each well, and the resulting agglomeration of AuNPs was assessed using a UV-Vis spectrophotometer (Ocean Optics UBS 2000, Florida, USA). Bioconjugation tests were conducted at room temperature under orbital agitation at 100 rpm.

### 2.4. Characterization of Nanomaterials

The morphology and size distribution of the nanoparticles were analyzed using a JEM-1400 transmission electron microscope (Jeol, Japan) operated at 120 kV with a tungsten filament. X-ray diffractometry was carried out with a scan in the 2θ region from 30° to 90°, 0.01° per minute, with a time constant of 2 s using a Phillips PW 1710 diffractometer with Cu ka radiation(Almelo, The Netherlands). UV-Vis absorption was read on a 200 to 800 nm scale with an Ocean Optics UBS 2000 spectrophotometer (Florida, USA), using ultrapure water for blank reading. Dynamic light scattering (DLS) and zeta potential (ZP) measurements were carried out using a Litesizer 500 (Anton Paar, Graz, Austria) device with 2 mL of the colloidal sample, and the final DLS values were expressed in nm and the zeta potential in mV. Infrared spectroscopy measurements were performed using an Agilent Cary 630 FTIR spectrometer (California, USA). Raman spectroscopy analysis was carried out using a MiraCal Methrom^®^ (Herisau, Switzerland) device with a spectral range of 400 to 2300 cm^−1^ using a 785 nm laser. The software used to process the data was OriginPro 8.5 SR1 and GraphPad Prism 6.

### 2.5. Detection Tests

For the detection tests, 594 blood serum samples were used; 387 were positive for anti-SARS-CoV-2 antibodies, and 207 were negative. The samples were obtained from Tommasi Labs and were registered with the Ethics Committee of CAAE/BRAZIL 51803621.1.0000.5060. The assays were carried out in 96-well plates where 50 µL of the AuNP nanoconjugate with spike protein and 50 µL of blood serum were distributed and maintained at room temperature for one hour, followed by detection conducted by Raman spectroscopy.

### 2.6. Database and Feature Extraction

The comprehensive database utilized the Raman spectra of all 594 blood serum samples. Due to the extensive information in the spectra (1901 points), various feature extraction and reduction techniques became necessary. These techniques included Principal Component Analysis (PCA) [24], Kernel Principal Component Analysis (kPCA) [25], t-distributed Stochastic Neighbor Embedding (t-SNE) [26], and Uniform Manifold Approximation and Projection (UMAP) [27]. In all cases, the features were reduced to only three, making it possible to visualize the samples in a three-dimensional plane formed by the transformed features.

### 2.7. Machine Learning

Machine learning investigations were carried out using the Scikit-Learn library and implemented in Python, and the characteristics of the Raman spectra of positive and negative SARS-CoV-2 samples were extracted using the UMAP technique, which showed the best separation of the samples. The machine learning models used were k-Nearest Neighbors (kNN) [28], SVM [29], Decision Trees (DT) [30], logistic regression (LR) [31], and Light Gradient Boosting Machine (LightGBM) [32]. A cross-validation technique, known as leave-one-out, was used to train and validate these algorithms. This strategy is suitable when there is a small database, and despite being computationally expensive, its results provide a reliable and unbiased estimate of the model’s performance [33].

## 3. Results and Discussion

### 3.1. Synthesis and Characterization of Gold Nanoparticles

**Figure 1:**
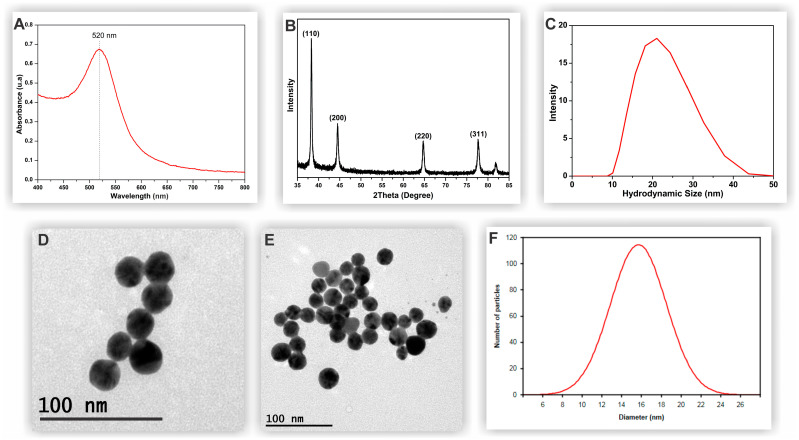
UV-Vis spectrum (**A**) and XRD pattern of gold nanoparticles (**B**). Histograms obtained using DLS (**C**). MET images of AuNPs (**D**,**E**). (**F**) Histogram of the size distribution of AuNPs obtained using MET (**F**).

The as-synthesized gold nanoparticles were confirmed by their very characteristic plasmon resonance peak at 520 nm (Figure 1A). Moreover, their crystal structure was determined by XRD and show four peaks at 38°, 31°, 44.45°, 64.64°, and 77.73° (Figure 1B), corresponding to the (111), (200), (220), and (311) planes of the crystal structure of the AuNPs [34,35]. DLS experiments (Figure 1C) showed that hydrated AuNPs had an average hydrodynamic size of 21.94 ± 0.77 nm, which is larger than the actual diameter owing to the presence of hydrated layers adhered to the surface. The ZP was −22.9 mV, indicating a high degree of stability. The analysis of MET images (Figure 1D,E) showed that the particle had a hexahedral shape, an average size of 15.47 ± 3.03 nm, and good monodispersion (Figure 1F).

### 3.2. Conjugation of Gold Nanoparticles with Spike Protein

Spike protein conjugation onto the surface of gold nanoparticles (AuNPs) was achieved through physical adsorption, where proteins electrostatically bind to the negatively charged AuNP surface due to the positive NH_2_ groups [20]. The optimal protein concentration for conjugation was determined using the gold number method, which identifies the minimum protein concentration required to prevent the salt-induced aggregation of nanoparticles [23]. Further details are provided in the Supplementary Materials, Figure S1.

**Figure 2:**
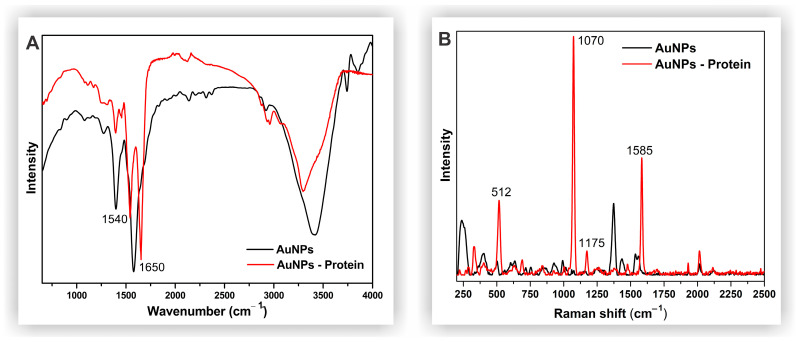
FTIR (**A**) and Raman (**B**) spectra of bare and protein-conjugated AuNPs.

In the FTIR analysis, spike protein was used to confirm its binding to the surface of the AuNPs. The spectra of the conjugated AuNPs (Figure 2A) exhibited a peak at 1650 cm^−1^, characteristic of primary amides, and a strong absorption peak at 1540 cm^−1^, attributed to the COOH group, confirming successful protein conjugation on the AuNP surface [23,36]. The SERS spectra of BSA conjugated with AuNPs revealed key interactions between the nanoparticles and the protein [37,38,39]. The band at 1585 cm^−1^ corresponds to the secondary amide region, while bands at 1175 cm^−1^ and 1070 cm^−1^ are indicative of C-N stretching. The band at 512 cm^−1^ is associated with S-S stretching in the disulfide bonds of cysteine [37,38,39]. Additionally, the bands between 1375 and 1600 cm^−1^ in the spectra of bare AuNPs correspond to the citrate capping agent, which stabilizes the nanoparticles [40,41].

### 3.3. Detection Tests

**Figure 3:**
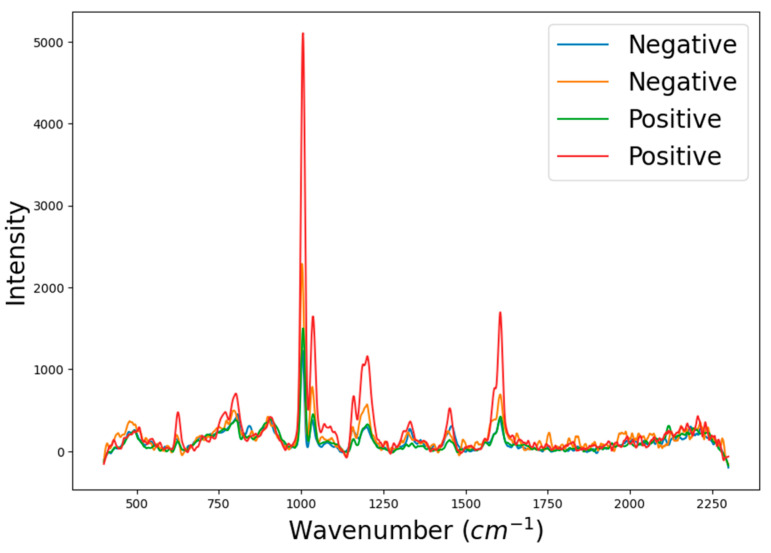
Raman spectra obtained from positive and negative samples for SARS-CoV-2 antibodies.

To collect data using the Raman spectroscope, the equipment was positioned vertically over the plate, with a 5 mm distance, ensuring that the laser was directed at the wells containing the samples. Figure 3 displays the spectra for positive and negative samples related to SARS-CoV-2 antibodies. Further details on sample acquisition are provided in Figure S2 of the Supplementary Materials.

When a positive SARS-CoV-2 sample interacts with AuNP@antigen, AuNP@antigen@antibody complexes form, leading to changes in the SERS signal. However, these changes may be subtle and challenging to distinguish in large datasets, complicating the identification of positive and negative samples. To address this challenge, the implementation of a machine learning approach was crucial in developing an effective analytical platform for point-of-care testing.

### 3.4. Machine Learning Approach

**Figure 4:**
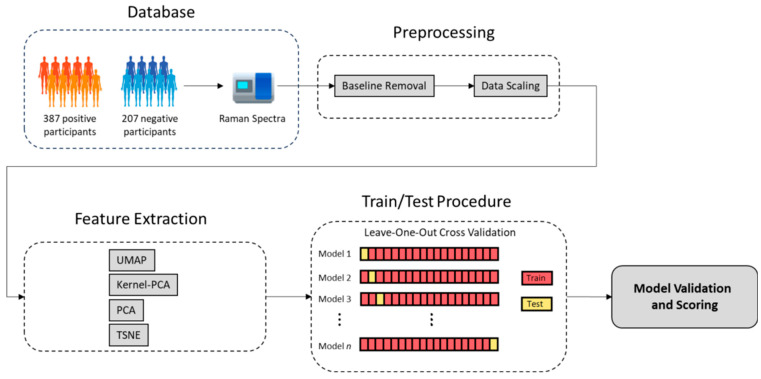
General machine learning pipeline for analyzing SERS data to distinguish SARS-CoV-2-positive and -negative participants.

Figure 4 illustrates a general machine learning pipeline designed for the analysis of SERS data obtained from participants who are either SARS-CoV-2 positive or negative.

The pipeline begins with the collection of SERS spectra from two groups of participants: 387 positive (indicated by the orange and red human-shaped figures) and 207 negatives (indicated by the blue human-shaped figures). These spectra are then stored in a digital format, ready for preprocessing.

The first step in preprocessing is baseline removal, which corrects any background noise or drift in the SERS data, ensuring that the spectra are more representative of the true signal. This is followed by data scaling (where zero mean and standard deviation equals one), a crucial step in the normalization of the data, allowing for better comparison between samples and enhancing the performance of the machine learning models.

Next, feature extraction is performed using several dimensionality reduction techniques, each reducing the high-dimensional SERS spectra to only three features. PCA is used to capture the most significant features by finding the directions (principal components) that maximize the variance in the data. Kernel PCA extends this by capturing non-linear relationships within the data. Additionally, t-SNE is used to convert similarities between data points into probabilities, which is useful for visualizing complex structures. Lastly, UMAP further reduces the data while preserving their most important characteristics. Each of these methods condenses the data into a simplified form with only three features, facilitating more effective model training.

Once the data are preprocessed, they are split into training and testing sets using the leave-one-out cross-validation (LOOCV) technique. This division allows the machine learning models to learn from the training data while being evaluated on unseen testing data to gauge their performance. The final step in the pipeline is model validation and scoring, where the models are assessed for their accuracy and generalizability in distinguishing between SARS-CoV-2-positive and -negative cases. This comprehensive pipeline ensures that the SERS data are accurately processed and analyzed, leading to reliable diagnostic models.

### 3.5. Feature Extraction

**Figure 5:**
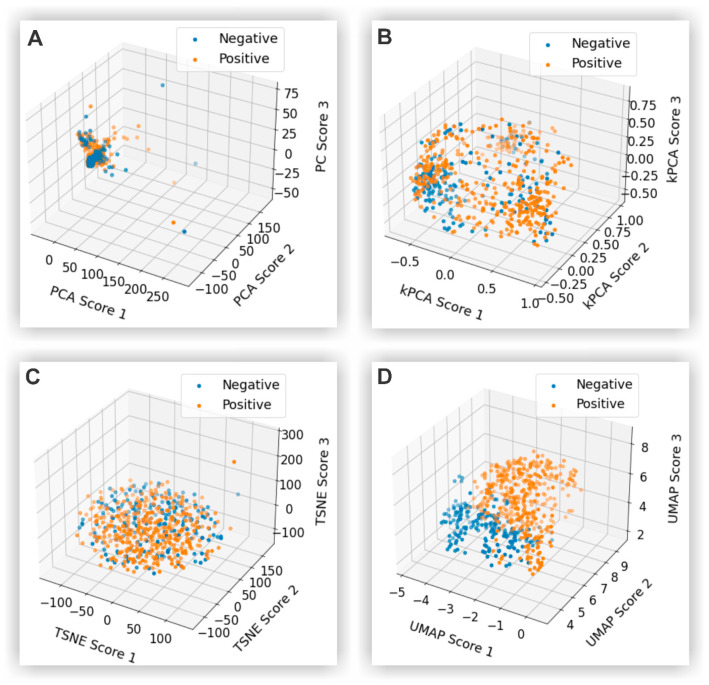
Feature reduction strategy using PCA (**A**), kPCA (**B**), t-SNE (**C**), and UMAP (**D**).

The feature extraction phase is crucial for reducing the high-dimensional SERS spectra data to a more manageable form, facilitating effective model training. The results of these techniques are illustrated in Figure 5, with each method presenting distinct characteristics.

Figure 5A shows that PCA effectively captures the most significant linear relationships within the data, providing a straightforward and interpretable reduction. However, PCA may not perform well with complex, non-linear data structures, limiting its ability to separate positive and negative samples distinctly.

kPCA (Figure 5B) extends PCA by capturing non-linear relationships through kernel functions. This technique provides a more nuanced representation of the data compared to PCA, allowing for better differentiation between positive and negative samples. Despite this improvement, kPCA still faces challenges in handling highly intricate data structures as effectively as some of the more advanced techniques.

t-SNE (Figure 5C) focuses on preserving the local structure of the data, converting similarities between data points into probabilities. t-SNE is particularly effective for visualizing complex structures and clusters within the data, making it a valuable tool for exploratory data analysis. However, t-SNE’s stochastic nature can lead to variability in the results, even with a fixed random seed, and it may struggle with scalability for very large datasets.

UMAP (Figure 5D) combines the strengths of preserving both local and global data structure, offering a balanced approach to dimensionality reduction. UMAP consistently achieved the best separation between positive and negative samples in this study, demonstrating its effectiveness in maintaining the essential characteristics of the data. Nevertheless, UMAP’s stochastic characteristics can introduce variability in the results, similar to t-SNE, but it generally outperforms the other techniques in this context [25].

Table 1 summarizes the key parameters used in each feature extraction algorithm, including kernels, output metrics, and other relevant settings. These parameters were selected by balancing data structure preservation, computational efficiency, and interpretability. Each algorithm relies on distinct parameters that influence performance. For instance, t-SNE and UMAP depend on visual or qualitative evaluations to assess how effectively clusters and patterns are maintained. Fine-tuning these dimensionality reduction algorithms involves carefully balancing the retention of data structure, processing efficiency, and result interpretability. Experimenting various parameter values and cross-validating through performance metrics or visual inspection was essential to achieving optimal results.

Overall, while each dimensionality reduction technique has its strengths and limitations, UMAP emerges as the most effective method for this study, providing the clearest distinction between SARS-CoV-2-positive and -negative samples.

### 3.6. Model Validation and Scoring

The model validation and scoring process evaluated five machine learning algorithms: kNN, SVM, DT, LR, and LightGBM. The hyperparameters for each of these algorithms were optimized using GridSearch to ensure the best possible configuration. The performance of the algorithms was assessed using LOOCV and various metrics, including accuracy, precision, sensitivity, F1-score, specificity, and efficiency. Table 2 and Figure S3 summarize the results of these evaluations, with the best results highlighted in bold.

Among the algorithms, kNN and LightGBM achieved the highest F1-scores, 0.9517 and 0.9410, respectively, indicating their superior ability to balance precision and recall. These two algorithms also showed high precision (kNN: 0.9605, LightGBM: 0.9338) and sensitivity (kNN: 0.9431, LightGBM: 0.9483), making them effective for detecting both positive and negative SARS-CoV-2 cases. The accuracy for kNN and LightGBM was also high, at 0.9377 and 0.9225, respectively, reflecting their overall reliability in classification tasks.

SVM performed moderately well, with an F1-score of 0.8422, precision of 0.8500, and sensitivity of 0.8346. Its accuracy was 0.7962, which is lower than that of kNN and LightGBM, but it still showed reasonable effectiveness with a specificity of 0.7246 and efficiency of 0.7796.

The DT algorithm demonstrated a good balance of metrics, achieving an F1-score of 0.9169, precision of 0.9065, and sensitivity of 0.9276. Its accuracy was 0.8905, with a specificity of 0.8212 and efficiency of 0.8744, indicating its capability to handle the classification task well.

LR had the lowest performance among the evaluated algorithms, with an F1-score of 0.8409, precision of 0.8222, and sensitivity of 0.8604. Its accuracy was 0.7878, and it showed the lowest specificity (0.6521) and efficiency (0.7563), likely due to its linear nature, which does not capture the non-linear characteristics of the problem as effectively as the other algorithms.

The confusion matrices in Figure S3 further illustrate the performance of these algorithms. The matrices show that kNN and LightGBM had the highest correct classifications for both positive and negative cases, while logistic regression had the highest number of misclassifications. The effectiveness metric, which considers both specificity and sensitivity, highlighted kNN (0.9353) and LightGBM (0.9113) as the top performers, emphasizing their robustness in distinguishing between positive and negative samples. Overall, the use of the UMAP algorithm for feature extraction significantly contributed to the high performance of these models.

To validate our findings, it is essential to compare the results obtained with those reported in the literature. Table 3 presents a comprehensive comparison with other papers, showcasing the kNN and LightGBM models.

The data in Table 3 demonstrate that the results obtained in this work are consistent with those reported in the literature. However, our study stands out in several key aspects. First, despite similarities in certain metrics, our model was developed using a larger dataset (employing blood serum samples), resulting in enhanced robustness and generalizability. Additionally, colloidal AuNPs simplify the methodology, offering easier sample preparation, rapid analysis, and improved accuracy, making it a more efficient and cost-effective alternative. In contrast, techniques such as mass spectrometry introduce a significant cost factor [14,41]. In this sense, the SERS approach described in this work minimizes these costs without sacrificing performance. These cited advantages, such as practicality, cost-efficiency, and scalability, differentiate our approach from existing studies.

To provide additional insights into the configuration of each machine learning algorithm, Table 4 summarizes the principal parameters used for each algorithm. These parameters were selected through GridSearch to identify the optimal configuration for each model, influencing their respective performances as reflected in the evaluation metrics.

## 4. Conclusions

This study demonstrated an effective approach for detecting SARS-CoV-2 using surface-enhanced Raman spectroscopy (SERS) data, focusing on feature extraction and machine learning algorithms. Among the dimensionality reduction techniques evaluated—PCA, kPCA, t-SNE, and UMAP—UMAP proved most effective in distinguishing between positive and negative samples by preserving both local and global data structures.

Five machine learning algorithms—k-Nearest Neighbors (kNN), Support Vector Machine (SVM), Decision Tree (DT), logistic regression (LR), and Light Gradient Boosting Machine (LightGBM)—were assessed. kNN and LightGBM emerged as the top performers, achieving high accuracy and sensitivity, supported by clear visualizations from UMAP. Logistic regression performed poorly due to its linear nature, which struggled with the complex, non-linear patterns in the data.

The results underline the value of combining advanced feature extraction with robust classification techniques for biomedical applications. This study also highlights areas for future research, such as optimizing model parameters and exploring hybrid approaches. Overall, this work sets a new benchmark for rapid and accurate SARS-CoV-2 detection using SERS data, with UMAP and kNN leading the way.

## Figures and Tables

**Table 1:** Best parameters for feature extraction algorithms.

Algorithms	Parameters	Description
PCA	n_components = 3	Number of principal components.
kPCA	kernel = “cosine”	Type of kernel function used.
t-SNE	perplexity = 10; metric = “chebyshev”	Perplexity measures the effective number of neighbors and metric specifies the distance metric to use when calculating pairwise distances between data points.
UMAP	n_neighbors = 7; min_dist = 0.6; output_metric = “chebyshev”; metric = “symmetric_kl”	Number of neighbors, minimum distance parameter, distance metric used in high-dimensional space, and distance metric used in low-dimensional space.

**Table 2:** Results obtained by machine learning algorithms using leave-one-out in the SARS-CoV-2 sample database.

Algorithms	Precision	Sensitivity	F1-Score	Accuracy	Specificity	Efficiency
kNN	**0.9605**	0.9431	**0.9517**	**0.9377**	**0.9275**	**0.9353**
SVM	0.8500	0.8346	0.8422	0.7962	0.7246	0.7796
DT	0.9065	0.9276	0.9169	0.8905	0.8212	0.8744
LR	0.8222	0.8604	0.8409	0.7878	0.6521	0.7563
LightGBM	0.9338	**0.9483**	0.9410	0.9225	0.8743	0.9113

**Table 3:** Comparison of the analytical performance of SERS combined with machine learning with other strategies for SARS-CoV-2 detection.

Technique	Model	Target	N° Samples	Sensitivity	Specificity	Accuracy	Ref.
MALDI FT-ICR MS	SVM	Proteins	97	1.0	0.875	0.956	[14]
MALDI-MS	SVM	Proteins	362	0.947	0.926	0.939	[42]
FTIR	kNN, PLS	-	243	0.87	0.66	78.4	[43]
FTIR	URF	-	265	0.93	0.83	0.85	[15]
SERS	LR, LDA, SVM	Antigen	46	-	-	0.96	[17]
SERS	kNN	Antibody	594	0.9431	0.9275	0.9377	This work
SERS	LightGBM	Antibody	594	0.9483	0.8743	0.9225	This work

Legend: URF = Unsupervised Random Forest, LDA = linear discriminant analysis.

**Table 4:** Principal parameters for machine learning algorithms.

Algorithms	Parameters	Description
kNN	n_neighbors = 7	Number of nearest neighbors considered.
SVM	kernel = “rbf” C = 1.0 gamma = “scale”	Type of kernel function, regularization parameter C, and kernel coefficient gamma.
DT	max_depth = 4 min_samples_split = criterion = “gini”	Maximum depth of the tree, minimum number of samples required to split an internal node, and function to measure the quality of a split.
LR	penalty = “l2” C = 1.0 solver = “lbfgs”	Norm used in penalization, inverse of regularization strength C, and algorithm to use in the optimization problem.
LightGBM	num_leaves = 31 learning_rate = 0.1 n_estimators = 100	Maximum number of leaves in one tree, step size of the learning rate, and number of boosting iterations.

## Data Availability

All data generated or analyzed during this study are included in the article and its Supplementary Materials. Any additional inquiries should be directed to the corresponding author.

## Supplementary Materials

The following supporting information can be downloaded at https://www.mdpi.com/article/10.3390/bios14110523/s1, Figure S1: Gold number of nanoparticles conjugated with Abcam spike protein, Figure S2: Illustration of data acquisition in a 96-well plate with a portable Raman spectrometer, Figure S3: Confusion matrices obtained by the (A) kNN, (B) SVM, (C) DT, (D) LR, and (E) LightGBM algo-rithms.
