# Supplementary material for: Surface-Enhanced Raman Scattering Combined with Machine Learning for Rapid and Sensitive Detection of Anti-SARS-CoV-2 IgG
Source: Biosensors (Basel). 2024 Oct 29;14(11):523. doi: 10.3390/bios14110523 (PMC11591781; doi:10.3390/bios14110523)
Supplement: Supplementary file 1 [file biosensors-14-00523-s001.zip › biosensors-3239079-supplementary.pdf]

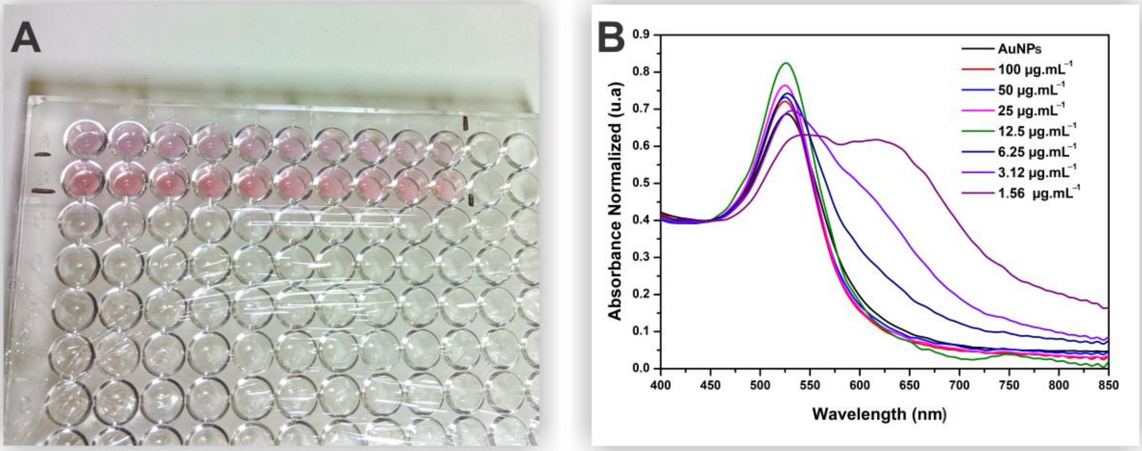

Figure S1. A) 96-well plate with the Gold Number experiment of nanoparticles conjugated with Abcam spike protein. B) UV-Vis spectra of the protein interaction with AuNPs

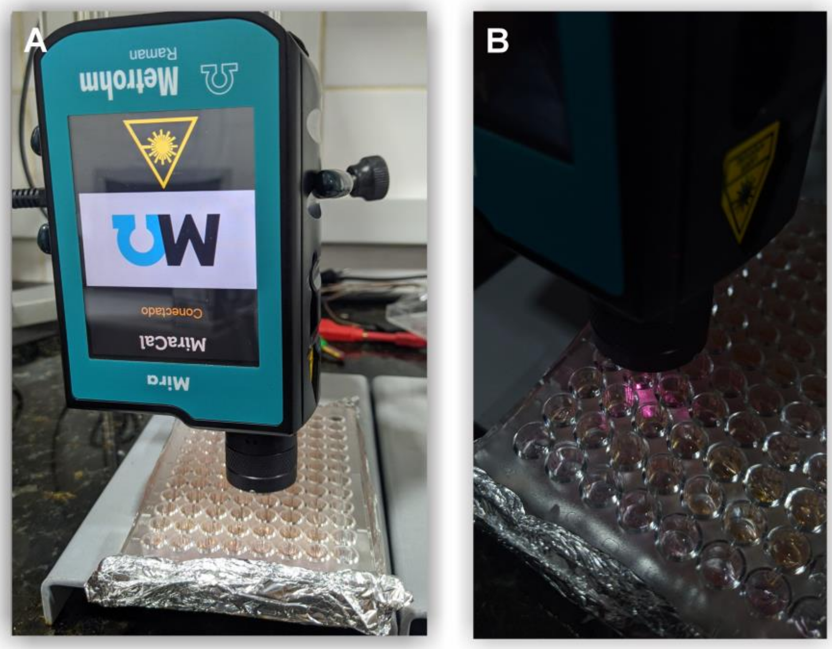

Figure S2. A) Illustration of the portable Raman spectrometer and the 96-well plate, B) How the data acquisition in a 96-well plate with a portable Raman spectrometer.

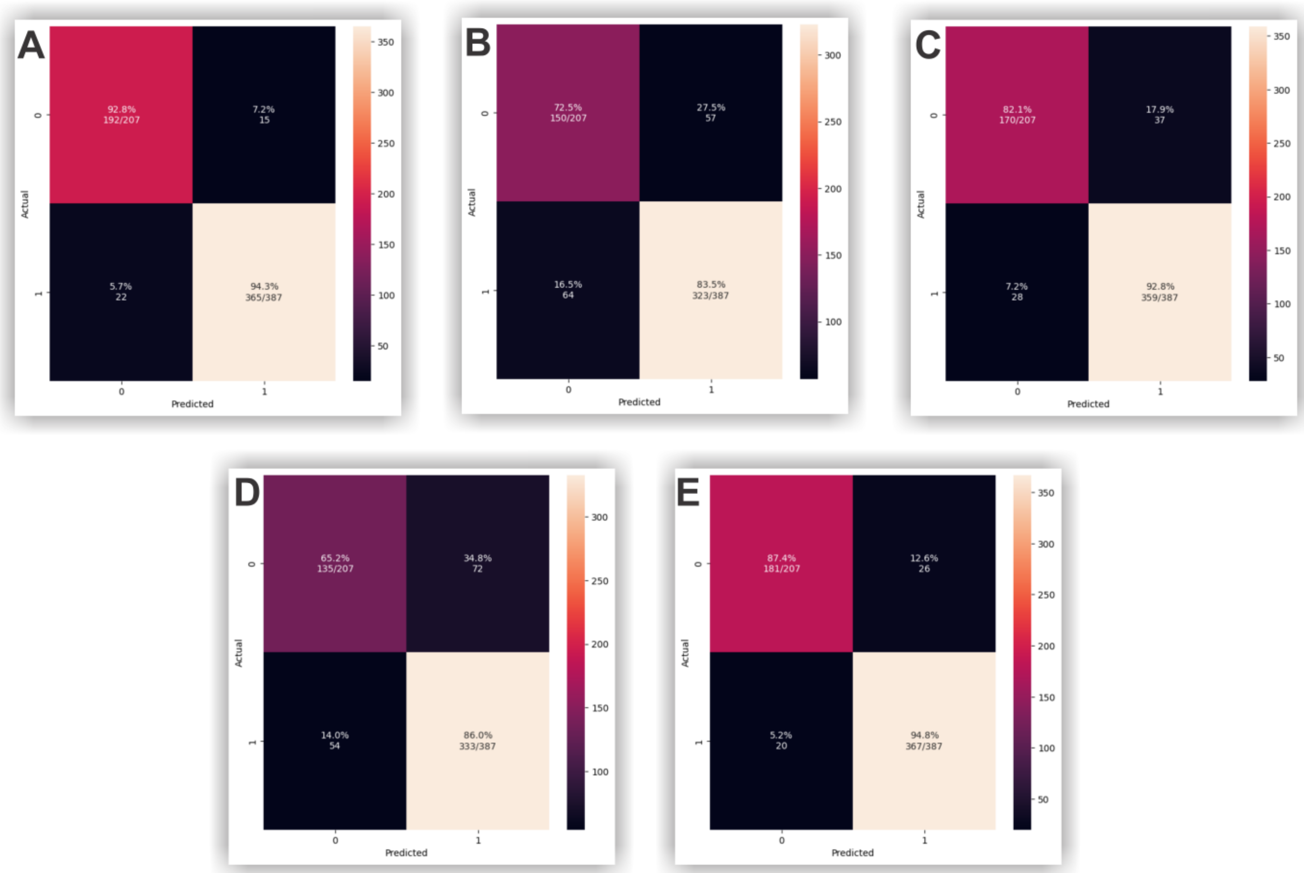

Figure S3. Confusion matrices obtained by the (**A**) kNN, (**B**) SVM, (**C**) DT, (**D**) LR, and (**E**) LightGBM algorithms.
